# Feasibility of Measuring Hair Glucocorticoids as a Potential Biomarker for Chronic Stress in Older Adults With Intellectual Disabilities

**DOI:** 10.1111/jir.70040

**Published:** 2025-09-09

**Authors:** Jasper Steven Dijkema, Mylène Nathalie Böhmer, Patrick Jan Eugene Bindels, Dederieke Anne Maria Maes‐Festen, Alyt Oppewal

**Affiliations:** ^1^ Department of General Practice, Intellectual Disability Medicine Research, Erasmus MC University Medical Center Rotterdam Rotterdam the Netherlands; ^2^ Department of General Practice, Erasmus MC University Medical Center Rotterdam Rotterdam the Netherlands

**Keywords:** aged, biomarkers, feasibility studies, hypothalamo‐hypophyseal system, physiological stress, psychological stress, vulnerable populations

## Abstract

**Background:**

Chronic stress can significantly impact health, leading to conditions such as cardiovascular disease and mental health issues. Detecting chronic stress in older adults with intellectual disabilities (ID) is challenging, but measuring scalp hair glucocorticoids (HairGC) may offer a solution. This study aims to investigate the feasibility of measuring HairGC in older adults with ID and assess reasons for failed sample collection and analysis.

**Methods:**

Hair samples were collected in the Healthy Ageing and Intellectual Disabilities (HA‐ID) cohort study (*n* = 278, 71.3 years [SD 6.2]). Feasibility was described as overall feasibility (percentage of successful measurements out of the total group) and quantified by consent rate (participants who consented for hair sample collection), collection rate (successfully collected hair samples from those who consented), and analysis rate (successfully analysed samples). Rates were categorised as low (< 25%), moderate (≥ 25–< 50%), good (≥ 50–< 75%) or excellent (≥ 75%), with stricter cutoffs for analysis rate (low: < 75%, moderate: ≥ 75–< 85%, good: ≥ 85–< 95%, excellent: ≥ 95%). Feasibility rates and reasons for failed collection and analysis were analysed for the total group and subgroups by age, sex and level of ID.

**Results:**

The feasibility of consent rate (204/278; 73%), collection rate (103/204; 50%) and analysis rate (89/103; 86%) was good. Overall, HairGCs were successfully measured for 89 out of 278 participants (32%), showing a moderate overall feasibility. Reasons for collection failure (*n* = 101/204) were hair that was too short or too thin (*n* = 65, 64%), resistance (*n* = 9, 9%), no‐shows (*n* = 5, 5%), other reasons (*n* = 9, 9%) and unknown reason (*n* = 13, 13%). Reasons for analysis failure (14/103) were not enough material (*n* = 12, 86%) and lost samples (*n* = 2, 14%). Overall feasibility rate was lower in males (15%) than in females (50%; *p* < 0.001) and higher in participants with moderate ID (42%) than in those with severe and profound ID (25%; *p* = 0.004).

**Conclusions:**

Overall feasibility of measuring HairGCs in older adults with ID was moderate. Feasibility was lower in males, with insufficient hair length/thickness as the main limitation, and higher in participants with moderate ID. HairGC measurement appears most feasible in females and less so in balding males, limiting its broader applicability as a stress measurement tool in an older population.

## 1 Background

Chronic stress is widely recognised as a significant contributor to conditions such as heart disease, diabetes and depression, highlighting the importance of measuring stress for effective prevention and early intervention (Keller et al. 2012; Lagraauw et al. 2015; Wright et al. 2015). The close link between chronic stress and disease is bidirectional, with various health conditions potentially inducing chronic stress, while chronic stress itself can adversely affect physical and mental health (Epel et al. 2018). Chronic stress is a prolonged state of stress resulting from ongoing stressors, such as psychological (e.g., relationship conflicts) or physiological (e.g., sleep deprivation), which leads to the persistence of the body's temporary stress response (Chu et al. 2024). While the definition of a prolonged state of stress varies in literature, a 3‐month period of a state of stress is commonly used as a benchmark (Wester and van Rossum 2015). Currently, several tools are available to measure the physiological manifestations of stress. For example, cortisol, a glucocorticoid regulated by the hypothalamic–pituitary–adrenal (HPA) axis, as well as adrenaline and inflammatory markers, can be measured as biomarkers of the body's stress response. Other tools include autonomic nervous system assessments, such as heart rate variability analysis, and advanced imaging techniques like functional MRI (Kim et al. 2018; Lever‐van Milligen et al. 2020; Pitman et al. 2012). These stress measurement instruments are still primarily used in research contexts to detect chronic stress rather than in health care organisations (Dorsey et al. 2022; Roberts and McWade 2021).

The need to integrate tools to measure chronic stress into healthcare is particularly pressing for older adults with intellectual disabilities (ID), who face unique challenges in health and ageing. Older adults with ID are known to experience a higher prevalence of somatic and mental conditions, as well as age‐related conditions at a younger age, compared to the general population (de Leeuw et al. 2022; McMahon and Hatton 2021; O'Dwyer et al. 2016). Additionally, the underdiagnoses of both somatic and mental diseases are commonly reported in this population (Bond et al. 2020; de Leeuw et al. 2022). Unfortunately, measuring chronic stress in older adults with ID presents specific difficulties. Obstacles that come along with invasive diagnostics, such as needle‐related anxiety, and the need for assistance in accessing healthcare services complicate medical and laboratory diagnostics (Bertelli et al. 2023; Mizen et al. 2012; Rava et al. 2023; Shea et al. 2022; Weise et al. 2021). Furthermore, limited communication and self‐reporting abilities make perceived stress measurement tools less suitable (de Witte et al. 2021; Maguire et al. 2023). Therefore, the availability of a low‐invasive method to measure chronic stress is particularly important for this vulnerable population.

A potential low‐invasive diagnostic tool for assessing chronic stress in healthcare is measuring glucocorticoid concentration in hair (HairGC) (Wester and van Rossum 2015). The collection of hair samples for HairGC measurement is noninvasive and accessible, as the sample is taken with scissors from the proximal scalp. Typically, 3‐cm hair segments are used to determine HairGC concentrations over 3 months, given the consistent growth rate of 1 cm per month (Abell et al. 2016; Ford et al. 2016; Noppe et al. 2015). Glucocorticoids diffuse from blood capillaries to hair follicles, where they integrate into the strands and remain stored for months (Heimbürge et al. 2020; Hodes et al. 2018; Stalder et al. 2017). HairGC thereby could quantify HPA activity by measuring cortisol (HairF) and its inactive metabolite cortisone (HairE) levels in hair, providing insight into long‐term stress levels (Iob and Steptoe 2019; Li et al. 2023; van der Valk et al. 2022). HairGC is increasingly used as a biomarker for chronic stress. Applications include assessing stress in caregivers, children and patients with psychiatric and somatic conditions. Kalliokoski et al. (2019) provide a systematic overview of these applications and highlight the potential of hair glucocorticoids as a stress biomarker, while also noting methodological inconsistencies and unresolved questions that currently limit its clinical implementation (Kalliokoski et al. 2019). Importantly, few studies to date have explored the feasibility or utility of HairGC measurement in people with ID, even though it may represent a promising tool for assessing chronic stress in this population. Given the communication challenges in this group, an objective biomarker such as HairGC could help assess chronic stress when self‐report measures are limited (de Witte et al. 2021; Maguire et al. 2023).

Previously, a pilot study reported successful hair sample collection for HairGC measurements in 17 of 26 adults with ID (65%). This small pilot study included adults with mild (14.3%), moderate (21.4%) and severe and profound ID (64.3%), with depressive symptoms (Hamers et al. 2021). Further research is needed to replicate these findings in a larger and more heterogeneous group. Therefore, this study aims to assess the feasibility of HairGC measurements in a large group of older adults with ID and additionally investigate reasons for failed hair sample collection and analysis. This will be done for the total group, and for subgroups based on age, sex and level of ID. Exploring the feasibility of HairGCs as a diagnostic tool for older adults with ID represents one of the first steps toward a possible integration of this method into ID healthcare practice.

## 2 Methods

### 2.1 Design and Participants

This study is a cross‐sectional study within the Healthy Ageing and Intellectual Disabilities (HA‐ID) multicentre cohort study, focusing on the health of older adults with ID. The HA‐ID study is an initiative of the Academic Collaborative Research Center HA‐ID, a partnership between Intellectual Disability Medicine Research Group, Department of General Practice of the Erasmus MC, University Medical Center Rotterdam, and the participating ID care organisations Abrona, Amarant and Ipse de Bruggen. The inception of the cohort dates from 2008, comprising 1050 older adults with ID aged 50 and above, who received care or support from one of the three participating organisations, which provide comprehensive, long‐term care and support tailored to individual needs. The participating organisations deliver care to a broad spectrum of individuals with ID (ranging from borderline to profound ID) across various settings, including centralised residential facilities, community‐based homes, day activity centres and supported living arrangements.

In the 10‐year follow‐up of the cohort, the collection of hair samples for HairGC measurement was introduced into the HA‐ID measurement protocol for the first time. All individuals (*n* = 429) remaining from the initial group of 1050 participants were invited to participate in this wave. For the consent procedure, those with the ability to understand the information provided, as evaluated by the behavioural expert of the ID care organisations, were asked for written informed consent themselves. For those incapable of making this decision, legal representatives were asked for written informed consent. Written informed consent for participation was obtained from 278 participants or their representatives. As part of the consent procedure, consent was additionally asked for the collection of hair samples. Data were collected from January 2020 to March 2023. More detailed information on the 10‐year follow‐up study protocol, including study design, participant recruitment, consent procedures and data collection procedures, is published elsewhere (de Leeuw et al. 2022).

### 2.2 Ethical Considerations

The Medical Ethics Review Committee Erasmus MC, University Medical Center Rotterdam approved the 10‐year follow‐up of the HA‐ID study. This study was designed and conducted in accordance with the ethical principles outlined in the Declaration of Helsinki (World Medical 2025).

### 2.3 Measurements

#### 2.3.1 Participant Characteristics

Information on age and sex (male/female) was gathered from the administration systems of the ID care organisations. Level of ID, categorised as borderline, mild, moderate, severe and profound, was extracted from the behavioural records by the behavioural expert of the ID care organisations. Additionally, HA‐ID staff collected information from medical records on the aetiology of ID (congenital, perinatal, acquired brain injury up to age 22 or unknown), residential status (central, community‐based, independently with ambulatory care, with family/close relations or unknown) and day care participation (yes/no). Professional caregivers of participants filled out questions about alcohol use (yes/no), tobacco use (yes, history of smoking or never), mobility (independent, with support or wheelchair), the use of corticosteroids (name, doses, frequency, location and duration) and whether hormone replacement therapy (oestrogen/progesterone for postmenopausal women) was provided in the 3 months preceding the date of hair sample collection. Furthermore, participants or their caregivers completed a hair sample collection questionnaire prior to hair sample collection, which included items with similar details on corticosteroid and hormone replacement therapy use.

#### 2.3.2 HairGC Measurement

Scalp hair of a minimum length of 3 cm and approximately the size of 300 hairs was cut from the vertex posterior, as close to the skin as possible, by trained HA‐ID staff using hair scissors. This method was in line with the Society of Hair Testing (SoHT) guidelines (Cooper et al. 2012). The hair sample was taped to a paper form, marking the proximal end, and stored in a paper envelope at room temperature. During storage, the first author (J.S.D.) measured the length of each hair sample using a tape measure to identify those shorter than the protocol‐specified minimum of 3 cm. All collected samples, regardless of their length, were sent to the laboratory of the Erasmus MC for analysis at the same time. For analysis, the proximal 3 cm of each collected hair sample was cut into three 1 cm segments. Each segment was then weighed to ensure it met the minimum required mass of 5 mg for successful analysis. If the segment met the minimum mass, methanol was added for sample extraction at 25°C. Following evaporation of the methanol, solid‐phase extraction was performed to selectively retain and subsequently elute the analytes, thereby purifying the sample. Cortisol and cortisone levels were then quantified using liquid chromatography–tandem mass spectrometry (LC–MS/MS). The analyses were performed using a validated laboratory protocol with established quality control procedures (Mirzaian et al. 2024). The results of the three 1‐cm segments were averaged, resulting in mean hair cortisol and cortisone concentrations (μg/mg) of the proximal 3 cm.

#### 2.3.3 Feasibility Quantification

The consent, collection and analysis rates were assessed to determine the overall feasibility of hair sample collection. The consent rate was defined as the percentage of participants who gave consent for hair sample collection, regardless of whether the collection was successful. The collection rate was defined as the percentage of participants who provided a hair sample, including those later found to be off‐protocol collections due to being too short (sample measuring < 3 cm) or too thin (segment weighing < 5 mg), among those who consented to hair sample collection. The analysis rate was defined as the percentage of hair samples successfully analysed out of all collected hair samples. Finally, the overall feasibility rate was calculated, representing the percentage of successfully analysed hair samples from participants who provided consent for the 10‐year follow‐up HA‐ID study. Consent, collection and overall feasibility rate were classified as low (< 25%), moderate (≥ 25% and < 50%), good (≥ 50% and < 75%) or excellent (≥ 75%), based on criteria from previous feasibility studies (Hilgenkamp et al. 2013; Weterings et al. 2020). For the analysis rate, a stricter classification was used: low (< 75%), moderate (≥ 75% and < 85%), good (≥ 85% and < 95%) and excellent (≥ 95%), to emphasise that lab analysis should not significantly impact overall feasibility once consent and collection are successful.

#### 2.3.4 Reasons for Failure

Reasons for failed hair sample collection were documented in the hair sample collection questionnaire. This included hair being too short or too thin (as assessed by HA‐ID staff prior to collection and analysis), resistance during collection or other reasons with open answer. Reasons for failed analysis were reported on lab reports (open answer). Reasons for no consent were not reported.

### 2.4 Statistical Analyses

Descriptive statistics were used to present participant characteristics as means (SD) for continuous variables and counts (percentages) for categorical variables, for the total group and the group with successfully analysed hair samples. Reports of corticosteroid use and/or hormone replacement therapy, both documented in medical records and in hair sample collection questionnaires, were included in the dataset. If discrepancies were found between the medical records and the questionnaire, the highest reported dose was used. Corticosteroid use was categorised based on the route of administration (systemic, scalp or other topical applications). Differences in participant characteristics between the total group and the group with successfully analysed hair samples were analysed with independent *t*‐tests (continuous variables) and Pearson's chi‐square tests (categorical variables). Categories comprising five or fewer individuals were not subjected to significance testing due to the increased risk of falsely detecting significant differences by chance (Type 1 error).

Feasibility rates and reasons for failed collection and analysis were analysed for the total group and subgroups by age, sex and level of ID. Feasibility rates and reasons for failed hair sample collection and analysis (*n*, %) were presented in a flow chart. For age, the study population was divided into three subgroups: 60 to 69 years, 70 to 79 years and those of 80 years and older. For level of ID, the categories borderline and mild ID and severe and profound ID were combined due to small subgroup numbers. Differences in feasibility rates and failed hair sample collection and analysis between subgroups based on age, sex and level of ID were assessed using Pearson's chi‐square tests. When significant differences were found across multiple groups, post hoc pairwise comparisons were conducted using Pearson's chi‐square tests to determine which specific groups differed. Subgroups comprising five or fewer individuals were not subjected to significance testing.

All statistical analyses were performed using the Statistical package of Social Sciences version 28.0 for Windows (IBM Corp, released 2021; IBM SPSS Statistics for Windows, version 28.0). Significance was set at *p* < 0.05.

## 3 Results

### 3.1 Participants Characteristics

A total of 278 participants provided consent for the 10‐year follow‐up HA‐ID study, with a mean age of 71.3 years (SD 6.2), consisting of 136 females (48.9%). The majority of participants had moderate (42.4%) or severe (28.1%) ID. Regarding residential status, most participants lived in central (57.6%) or community‐based (39.9%) settings. More details about the participant characteristics and differences between the total group and the group with successfully collected and analysed hair samples are presented in Table 1.

**TABLE 1:** Participants characteristics for the total group and participants with successfully analysed hair samples.

	Total group; *n* = 278	Participants with successfully analysed hair samples; *n* = 89	*p*
Age, mean (SD)	71.3 (6.2)	70.8 (6.2)	0.308
Sex (M/F) (*n*, %)	142 male (51.1%); 136 female (48.9%)	21 male (23.6%); 68 female (76.4%)	< 0.001*
Level of intellectual disability (*n*, %)			
Borderline	6 (2.2%)	0 (0.0%)	0.008*
Mild	40 (14.4%)	12 (13.5%)	
Moderate	118 (42.4%)	49 (55.1%)	
Severe	78 (28.1%)	21 (23.6%)	
Profound	34 (12.2%)	7 (7.9%)	
Unknown	2 (0.7%)	0 (0.0%)	
Aetiology of ID (*n*, %)			
Congenital	36 (12.9%)	13 (14.6%)	0.449
Down syndrome	19 (6.8%)	8 (9.0%)	
Fragile X syndrome	3 (1.1%)	2 (2.2%)	
Other syndromes	14 (5.0%)	3 (3.4%)	
Perinatal	14 (5.0%)	7 (7.9%)	
Acquired brain injury (up to age 22)	19 (6.8%)	8 (9.0%)	
Unknown	209 (75.2%)	61 (68.5%)	
Residential status (*n*, %)			
Central	160 (57.6%)	44 (49.4%)	0.220
Community based	111 (39.9%)	43 (48.3%)	
Independently with ambulatory care	1 (0.4%)	0 (0.0%)	
Family/close relations	0 (0.0%)	0 (0.0%)	
Unknown	6 (2.2)	2 (2.2)	
Daytime activity (*n*, %)			
Yes	171 (61.5%)	68 (76.4%)	**
No	15 (5.4%)	3 (3.4%)	
Unknown	92 (33.1%)	18 (20.2%)	
Alcohol use (*n*, %)			
No	161 (57.9%)	62 (69.7%)	0.996
Yes	26 (9.4%)	10 (11.2%)	
Unknown	91 (32.7%)	17 (19.1%)	
Tobacco use (*n*, %)			
Never	149 (53.6%)	58 (65.2%)	0.781
History of smoking	15 (5.4%)	5 (5.6%)	
Yes	25 (9.0%)	9 (10.1%)	
Unknown	89 (32.0%)	17 (19.1%)	
Mobility (*n*, %)			
Independent	94 (33.8%)	31 (34.8%)	0.511
Support	42 (15.1%)	17 (19.1%)	
Wheelchair	22 (7.9%)	10 (11.2%)	
Unknown	120 (43.2%)	31 (34.8%)	
Medication use (*n*, %)			
Corticosteroids	16 (5.8%); 2 systemic, 3 scalp, 11 other topical application	7 (7.9%); 1 systemic, 3 scalp, 3 topical others	**
Hormone replacement therapy	0 (0.0%)	0 (0.0%)	

*Note:* *p < 0.05; ** category comprising of five or fewer individuals, not subjected to significance testing.

Abbreviation: SD, standard deviation.

### 3.2 Consent Rate

**FIGURE 1:**
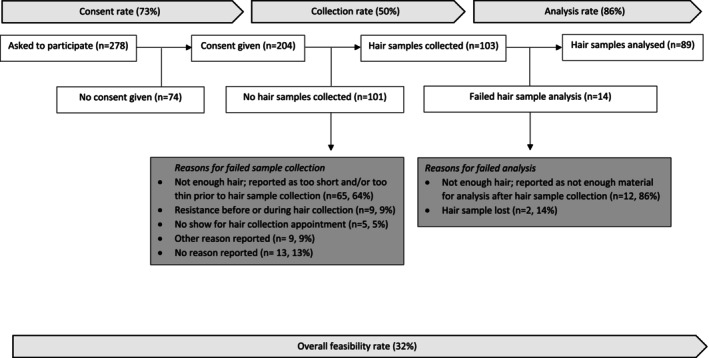
Flow diagram of measuring HairGCs.

Consent for hair sample collection was obtained from 204 out of 278 participants (73%), indicating a good consent rate (Figure 1, Table 2). Subgroup analyses revealed that the oldest age group (age ≥ 80 years) had a significantly lower consent rate (55%) than participants of 60–69 years (78%; *p* = 0.008). No significant differences in consent rate were found for sex and level of ID.

**TABLE 2:** Subgroup analysis of feasibility rates and reasons for failed hair sample collection.

	*n*	Feasibility rates				Reasons for failed sample collection				
		Overall feasibility rate	Consent rate	Collection rate	Analysis rate	Hair too short or too thin	Resistance	No show	Other reason	No reason
Total group	278	89/278 (32%)	204/278 (73%)	103/204 (50%)	89/103 (86%)	65/101 (64%)	9/101 (9%)	5/101 (5%)	9/101 (9%)	13/101 (13%)
Age										
60–69 years	125	46/125 (37%)	97/125 (78%)*, ^a^	53/97 (55%)	46/53 (87%)	29/44 (66%)	3/44 (7%)	2/44 (5%)	4/44 (9%)	6/44 (14%)
70–79 years	120	34/120 (28%)	89/120 (75%)*	41/89 (46%)	34/41 (83%)	31/48 (65%)	5/48 (10%)	3/48 (6%)	2/48 (4%)	7 (15%)
≥ 80 years	33	9/33 (27%)	18/33 (55%)*, ^a^	9/18 (50%)	9/9 (100%)	5/9 (56%)	1/9 (11%)	0/9 (0%)	3/9 (33%)	0/9 (0%)
Sex										
Male	142	21/142 (15%)*	105/142 (74%)	28/105 (27%)*	21/28 (75%)*	56/77 (73%)*	4/77 (5%)	5/77 (7%)	6/77 (8%)	6/77 (8%)
Female	136	68/136 (50%)*	99/136 (73%)	75/99 (76%)*	68/75 (91%)*	9/24 (38%)*	5/24 (21%)	0/24 (0%)	3/24 (13%)	7/24 (29%)
Level of Intellectual Disability										
Mild	46	12/46 (26%)*	33/46 (72%)	14/33 (42%)*	12/14 (86%)	13/19 (68%)	0/19 (0%)	2/19 (11%)	3/19 (16%)	1/19 (5%)
Moderate	118	49/118 (42%)*, ^b^	88/118 (75%)	58/88 (66%)*, ^b^	49/58 (84%)	20/30 (67%)	2/30 (7%)	1/30 (3%)	3/30 (10%)	4/30 (13%)
Severe/profound	112	28/112 (25%)*, ^b^	83/112 (74%)	31/83 (37%)*, ^b^	28/31 (90%)	32/52 (62%)	7/52 (14%)	2/52 (4%)	3/52 (6%)	8/52 (15%)

* *p* < 0.05; superscript letters indicate post hoc pairwise comparisons.

^a^ Significant difference between 60 to 69 years and ≥ 80 years.

^b^ Significant difference between moderate and severe/profound ID.

### 3.3 Collection Rate

Of the 204 individuals who provided consent, 103 samples were collected (50%), falling only just into the category of a good collection rate (Figure 1, Table 2). Subgroup analyses showed that males had a significantly lower collection rate than females (27% vs. 76%; *p* < 0.001). Furthermore, the collection rate was significantly higher in participants with moderate (66%) than in participants with severe/profound ID (37%; *p* = 0.001). No significant differences in the collection rate were found for age. The most common reason for failed hair sample collection was participants having too short or too thin hair, accounting for 65 out of 101 cases (64%). Additional reasons were resistance (9%), no‐shows (5%), other reasons (9%) and unknown reason (13%). Reasons for failed sample collection are shown in Figure 1. In terms of sample length, of all 103 samples collected, 10 samples (10%) were shorter than the 3 cm specified in the collection protocol.

### 3.4 Analysis Rate

Of the 103 collected samples, 89 samples were successfully analysed (86%), reflecting a good analysis rate (Figure 1, Table 2). Subgroup analyses showed that males had a significantly lower hair sample analysis rate compared to females (75% vs. 91%; *p* = 0.039). No significant differences in analysis rate were found for age and level of ID. Two samples (14%) were lost during transport and could therefore not be analysed (Figure 1). The primary reason for failed hair sample analysis was the collection of not enough hair material (< 5 mg), which occurred in 12 out of the 14 cases (86%). No analyses failed due to poor sample quality or assay performance.

### 3.5 Overall Feasibility Rate

Of the total group, HairGCs were successfully collected and analysed in 89 of the 278 participants who provided consent (32%), reflecting a moderate overall feasibility rate (Figure 1, Table 2). Subgroup analyses showed that males had a significantly lower overall feasibility rate than females (15% vs. 50%; *p* < 0.001). Also, participants with moderate ID had a significantly higher overall feasibility rate than participants with severe/profound ID (42% vs. 25%; *p* = 0.004). No significant differences in overall feasibility were found for age.

## 4 Discussion

We evaluated the feasibility of hair sample collection for measuring long‐term HairGCs in older adults with ID, focusing on consent, collection, analysis and overall feasibility rates. Our study found a moderate overall feasibility rate (32%), with a good consent rate (73%), a collection rate falling just into the good category (50%) and a good analysis rate (86%). Subgroup analyses indicated lower overall feasibility, and lower collection and analysis rates in males than in females, predominantly due to having too short or thin hair. Participants with moderate ID had significantly higher overall feasibility and collection rates than participants with severe/profound ID. Additionally, the oldest age group (age ≥ 80 years) had a significantly lower consent rate than participants aged 60–69 years.

Our findings align with a previous pilot study where HairGC measurement was first explored in adults with ID and depressive symptoms (Hamers et al. 2021). That study, including a younger sample (mean age 51.8, SD 9.4), reported a 65% overall feasibility rate, with ‘hair too short or too thin’ being the reason for five of the nine failed collections. In our older sample, the overall feasibility was lower (32%), and ‘hair too short or too thin’ was a more frequent issue (64%). Our findings build on the previous pilot study by confirming too short or too thin hair as a key challenge in a larger, more representative ID sample without depression. Additionally, we showed that sex (lower feasibility in males) and level of ID (higher feasibility in older adults with moderate ID) influenced the feasibility of HairGCs measurement in older adults with ID.

Fewer hair samples were collected from males, who showed low collection rates compared to good collection rates in females. This discrepancy may be attributed to factors such as age‐related hair loss, male pattern baldness or common male hairstyles, as supported by the higher frequency of ‘hair too short or too thin’ as a reason for failed hair collection in males. Interestingly, no differences were found among age subgroups regarding ‘hair too short or too thin’, likely because all participants were at an age prone to hair loss (Villani et al. 2022). The consent rate, however, was significantly affected by age, with individuals of 80 years and older and/or their legal representatives being less willing to provide consent. This finding might reflect a reluctance to engage in additional study procedures in the older age group, or their own awareness of having little or no hair, but further research is needed to clarify this. Regarding level of ID, participants with moderate ID showed higher feasibility and collection rates than those with severe/profound ID. We observed an absolute higher resistance to the hair sample collection in the severe/profound group (14%) versus the moderate ID group (7%), suggesting more difficulties with compliance or discomfort for this group. Due to the small sample size, statistical significance was not tested for reasons for failed collection, limiting our ability to draw conclusions about this. Larger studies are needed to confirm these findings and explore contributing factors.

These findings indicate HairGC measurement may be more suitable for females and less suitable for balding males, consistent with previous research in the general population (Fischer et al. 2017). This raises concerns about the practicality of using hair samples to measure HairGC in an older population and underscores the need for alternative methods for measuring long‐term glucocorticoids in balding males. Unless shorter and/or thinner hair segments prove effective for HairGC analysis, these limitations are likely to persist. Establishing the validity of using 1 cm hair segments, in addition to the commonly used 3 cm, could improve the feasibility of collecting hair samples in an older population. However, using shorter segments may affect the validity of HairGC as a biomarker for long‐term stress, as 1 cm of hair growth reflects only 1 month of stress exposure. Still, from a conceptual perspective, a month of persistent stress could still be considered long‐term or chronic, as its lived experience may not feel as transient as the time frame implies.

Although 1‐cm segments may still provide a valuable measurement, it is noteworthy that 10% of the samples in our study were cut too short, despite the protocol clearly specifying 3 cm. This deviation suggests that collectors may have either prioritised obtaining some hair over strictly following the protocol or misjudged the required length. To prevent unnecessary burden on participants, it is important to emphasise that adherence to the specified length is essential, as inadequate samples may be unusable.

### 4.1 Strengths and Limitations

This is the first study to measure HairGC in a relatively large sample of older adults with ID, who could benefit substantially from low‐invasive diagnostics for chronic stress. By prioritising feasibility, the needs and limitations of this group are considered early in the process of developing new stress measurement tools. Furthermore, our approach holds promise for incorporating this measurement into our HA‐ID cohort study, enabling the collection of valuable longitudinal data on stress. This integration could enhance our understanding of stress trajectories of older adults with ID over time.

Our study had some limitations. Although the initial HA‐ID cohort was near‐representative for older adults with ID receiving formal care and support (Hilgenkamp et al. 2011), those who consented to participate in this study twice may be more inclined toward research participation than the broader population, introducing potential selection bias. Additionally, feasibility was higher among individuals with moderate ID compared to those with severe or profound ID, which may further limit the generalizability of our findings. Furthermore, feasibility and consent rates for hair sample collection may be higher in younger individuals with ID, warranting further investigation in that group.

In the context of ID studies, the feasibility rates observed in our study may have been supported by the participating organisations' familiarity with research through their involvement in the HA‐ID cohort, as well as the possibility of person‐centred scheduling of hair sampling within daily care routines. In contrast, settings with less exposure to research activities or more rigid care routines may experience reduced feasibility. However, it is important to note that the main reason for failure in our study was the absence of sufficient hair for sampling, which is an age‐related factor that is unlikely to be influenced by contextual aspects. These contextual factors warrant consideration when interpreting and comparing the feasibility of hair glucocorticoid measurement across different countries or populations.

### 4.2 Future Research Directions

To improve feasibility in future studies, particularly among males and individuals with severe or profound ID, strategies such as (1) involving familiar caregivers to assist with hair collection, (2) using culturally sensitive and gender‐appropriate approaches to address reluctance regarding hair sampling and (3) providing visual aids or desensitisation protocols to increase acceptance among individuals with sensory sensitivities or anxiety should be explored.

In addition, future research should explore adaptation in analysis methods to reliably detect HairGCs from less hair material, thereby enhancing feasibility in older adults with ID. Investigating why participants refuse hair sampling could further refine protocols. Additionally, studies in younger individuals with ID, where feasibility may be higher, are recommended. Furthermore, expanding the HA‐ID cohort with longitudinal hair sampling to analyse trends in HairGC values over time, and comparing these findings with large‐scale studies in the general population, would provide greater insight into HairGC levels of older adults with ID and the potential clinical implications. Finally, it is essential to validate HairGCs as a biomarker for chronic stress in the ID population to determine its potential for clinical applications in ID healthcare.

## 5 Conclusions

Overall, the feasibility of hair sample collection for HairGC measurement in older adults with ID was moderate. The observed differences in feasibility rates and reasons for failed sample collection and analysis among subgroups suggest that the feasibility of hair sample collection and analysis in older adults with ID is notably influenced by sex (lower feasibility in males) and level of ID (higher feasibility in moderate ID). The primary limitation appeared to be insufficient hair length or thickness. Consequently, HairGC measurement seems most feasible in a selective group, primarily females, and less feasible in balding males, raising concerns about its applicability as a stress measurement tool in an older population.

## Conflicts of Interest

The authors declare no conflicts of interest.

## Data Availability

Data supporting the findings are available from the corresponding author upon reasonable request, with restrictions due to privacy and ethics.
